# Obituary

**Published:** 1880-12

**Authors:** 


### OBITUARY

Died.—On Sabbath, November 21st, Dr. Thomas Wood, of this city, by blood poisoning, contracted while giving professional attention to the victims of a railroad accident.

Dr. Wood was sixty-six years of age, and leaves Dr. Mussey, whose senior he was, the sole survivor of the surgeons of his day, the cotemporaries of Blackman—a group that made Cincinnati and her colleges famous the world over.

In early life he lectured in the Medical College of Ohio, and at one time was Professor of Anatomy in that institution. He was also, for a time, Professor of Anatomy in the Ohio College of Dental Surgery. Since that time he has served several terms as Chief Surgeon of the staff of the Cincinnati Hospital.

Dr. Wood, though naturally of a diffident and retiring nature, occupied during nearly the whole of his professional life some public position of honor and trust.

As a surgeon he had no superior in the West, and perhaps not in this country. His fame in this respect was national. He will be missed wherever he was known; and it is safe to say that few men could be more poorly spared to the community or to the profession than Dr. Wood. Personally he was quiet, social and benevolent. His charities were many; but in this respect "his right hand knew not what his left-hand did."

### OBITUARY.

Died.—At Madison Wisconsin, on Tuesday November 23d, Professor James C. Watson, in his forty-third year. For his attainments in general science—and especially of astronomy—he had a world wide reputation. Wherever astronomy is known and studied, Dr. Watson's name was familiar.

He was a large contributor to the literature of science. He added fame and history to that Institution, "The University of Michigan," with which he spent nearly his whole life, and probably did more than any other man to build for it a world-wide reputation. In his passing away the world sustains a great loss. Such men only appear at long intervals.

Those who knew him best will miss him most, for he was a greatly esteemed man.

